# Effect of Yoga on Blood Pressure in Prehypertension: A Systematic Review and Meta-Analysis

**DOI:** 10.1155/2021/4039364

**Published:** 2021-09-13

**Authors:** Janhavi Sandeep Khandekar, Vanamala Lakshmi Vasavi, Vijay Pratap Singh, Stephen Rajan Samuel, S. G. Sudhan, Bidita Khandelwal

**Affiliations:** ^1^Department of Physiotherapy, Kasturba Medical College, Mangalore, Manipal Academy of Higher Education, Manipal, India; ^2^Sikkim Manipal University, Sikkim Manipal Institute of Medical Sciences, Gangtok-737102, India

## Abstract

**Introduction:**

Prehypertension is a precursor for developing hypertension and is a risk factor for cardiovascular diseases. Yoga therapy may have a role in lowering the blood pressures in prehypertension and hypertension. This systematic review aims to synthesize the available literature for the same. *Methodology*. Databases such as PubMed, Embase, Scopus, and Web of Science were searched for randomised control trials only in the time duration of 2010–2021. The main outcome of interest was systolic and diastolic blood pressures. Articles were screened based on the inclusion criteria, and 8 articles were recruited for the review. Meta-analysis was done for suitable articles. RevMan 5.4 by Cochrane was used for meta-analysis and forest plot construction. Risk of bias was determined using the Downs and Black checklist by three independent authors.

**Results:**

The meta-analysis of the articles favoured yoga intervention over the control intervention. Yoga therapy had significantly reduced the systolic pressure (−0.62 standard mean difference, at IV fixed 95% CI: −0.83, −0.41) and diastolic pressure (−0.81 standard mean difference, at IV random 95% CI: −1.39, −0.22). Secondary outcome measures studied were heart rate, weight, BMI, waist circumference, and lipid profile. The main protocol of yoga therapy included postures, breathing exercises, and different meditation techniques. A significant reduction in secondary outcomes was observed, except for HDL values in lipid profile which showed a gradual increase in yoga group in comparison with alternative therapy.

**Conclusion:**

Yoga therapy has shown to be significant in the reduction of systolic and diastolic pressure in prehypertensive population. Supporting evidence lacks in providing a proper structured dosage of yoga asanas and breathing techniques. Considering the existing literature and evidence, Yoga therapy can be used and recommended in prehypertensive population and can be beneficial in reducing the chances of developing hypertension or cardiovascular diseases.

## 1. Introduction

Prehypertension and hypertension are one of the treatable diseases in the world. There has been strong evidence on the progression of prehypertension to hypertension, provided by the American Heart Association (AHA) in 2011. One of the studies also gives a probability of prehypertensive adults progressing to hypertension [1]. Prehypertension is defined as systolic blood pressure (SBP) 120–129 mmHg and diastolic blood pressure (DBP) 80–89 mmHg by the 2017 guidelines of AHA [2]. According to the update on the 8th guideline by Joint National Committee (JNC), it was SBP 120–139 mmHg and DBP 80–89 mmHg [1]. Prehypertension is a sign and can give the probability of developing cardiovascular diseases in the future. The Framingham heart study (FRS) has found the epidemiology for developing cardiovascular diseases and has identified elevated cholesterol levels and blood pressures as the important predisposing factors [3]. Elevated stress levels have also been correlated with a rise in blood pressure [4, 5]. Yoga improves flexibility, reduces stress levels, and causes strengthening of muscles. The neurobiological causes for increased stress levels were incorporated in a systematic review by Pascoe et al. in 2017 [6]. This systematic review and meta-analysis included the articles which used MBSR and yoga therapy in reducing stress levels and studied its physiological effects. This review did not solely concentrate on elevated blood pressures as a main outcome measure.

Yoga therapy may prove to be beneficial in hypertensive and prehypertensive population. There was a significant effect of yoga on hypertensive population [7]. As per the review by Park and Hans, yoga therapy and meditation are successful in reducing the systolic and diastolic blood pressures [8]. Yoga therapy has been proven to be more effective in comparison to meditation. This review has focused on both hypertensive and prehypertensive population and has not isolated prehypertension as the primary health condition. Supporting literature also has been found on both hypertension and prehypertensive population [7, 8]. Yoga therapy is proven to be beneficial in reducing the cardiovascular risks as per the review by Chu et al. in 2016 [9]. This review has included all the predisposing comorbidities for developing cardiovascular disease and not solely elevated blood pressures. A review solely focusing on prehypertension was not found.

Therefore, this review aims at providing evidence for stand-alone effect of yoga on prehypertensive population. To ensure high level evidence, this review will also aim to provide a meta-analysis for the blood pressure, systolic and diastolic.

## 2. Methodology

### 2.1. Literature Search

**Figure 1:**
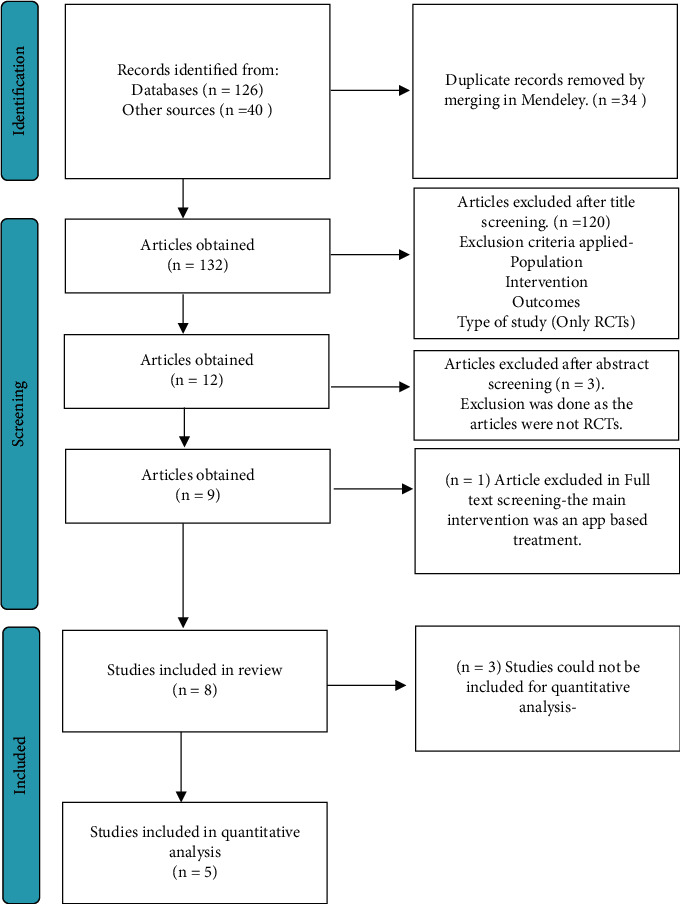
Prisma flow chart.

The protocol of this systematic review was registered in Open Science Framework (OSF) with the registration DOI: 10.17605/OSF.IO/YH2FQ. MEDLINE, Scopus, EMBASE, and Web of Science were screened, and searches were run using various search strategies with a combination of Booleans, AND and OR, separately and later combined to get the desired articles as shown in Table 1 through the search engines of PubMed and Embase. The articles which were unsuitable according to inclusion criteria were excluded. Inclusion criteria and exclusion criteria are given in the following. A total of 126 articles were shortlisted based on the various filters of databases mentioned above and selected for title and abstract screening. 40 articles were identified from sources other than the databases referred to above. After title, abstract and full-text screening, eight appropriate articles were finalised and taken for the systematic review as shown in Figure 1, and then they were reviewed. Synonyms and MeSH terms were identified using Cochrane and PubMed MeSH finders and search strategy builders. The synonyms which were used are described in Table 2.

### 2.2. Inclusion Criteria

- Studies with all forms of yoga, pranayama, and meditation
- Studies published in English language journals, human trials, and indexed in the databases mentioned above
- Types of studies: only randomised control trials (RCTs)

### 2.3. Exclusion Criteria

The exclusion criteria were as follows:

- Studies on aromatherapy, music therapy and cognitive behavioural therapy, speech therapy, and any gadget-based meditation techniques
- Studies involving tai chi, qi gong, or other types of such topics
- Studies involving other types of breathing other than pranayama or yogic breathing
- Type of studies: qualitative studies, cross-sectional studies, systematic review, case studies, nonrandomised clinical trials, and point of view
- Main outcomes: systolic and diastolic blood pressures
- Secondary outcomes: lipid profile, heart rate, BMI, and waist circumference

### 2.4. Data Extraction

The data extraction was done by 3 investigators simultaneously. The data were extracted using mean and standard deviation for each of the obtained articles. In articles where mean and standard difference was not available, mean and standard error or the mean difference was considered valid and extracted for suitable outcome measures. SBP and DBP were the primary outcomes. Secondary outcomes such as lipid profile (HDL, LDL, VLDL, TC, and triglycerides), waist circumference, BMI, heart rate, and weight were also taken in terms of mean and standard deviation/error/difference. The major time points of interest were pretest baseline characteristics and posttest on completion of duration of protocol.

### 2.5. Data Analysis

The obtained articles were studied for the main outcome measures. The outcome of interest was sought, and statistical values for the same were noted. The values of SBP and DBP were taken in terms of mean and standard deviation and, if available, mean difference. The reduction in the values of systolic and diastolic blood pressures were compared in pre- and postintervention groups in both the arms of each trial and the mean difference was computed only for the primary outcomes of interest. The values of secondary outcomes of interest were also noted and were analysed for pre-post changes in values.

**Figure 2:**
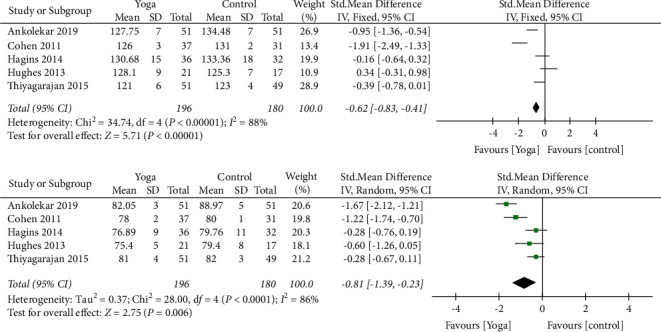
Forest plot: meta-analysis.

The scope of meta-analysis was identified in the primary outcomes of interest. Since all the outcomes were continuous, the mean difference for treatment effect was computed. Meta-analysis was done for primary outcomes of interest, that is, SBP and DBP, due to similarity in terms of the population, intervention, comparison, outcomes (PICO), and study design for the relevant data. The random-effects model was used for the meta-analysis because considerable heterogeneity was expected among the studies. The heterogeneity among the chosen studies was evaluated using the Chi^2^ statistic (*p* < 0.01 considered statistically significant), and heterogeneity was evaluated with the *I*^2^ statistic (>60% considered substantial heterogeneity). Meta-analysis was done using RevMan 5.4 software by Cochrane. The forest plots for meta-analyses of SBP and DBP have been presented in Figure 2. For other variables, a descriptive analysis has been made based on mean differences pre- to postintervention. However, this study aims to know changes in SBP and DBP after yoga intervention.

### 2.6. Outcome Measures

For SBP, five studies were analysed for FI level. 196 samples were present in yoga group and 180 in control group. Heterogeneity [*I*^2^] was 88% (pHeterogeneity <0.0001). The mean difference was −0.62 with ((95% confidence Interval) −0.83 to −0.41) for the intervention versus control group.

For DBP, heterogeneity [*I*^2^ ] was 86% (pHeterogeneity <0.0001). The mean difference was −0.81 (95% confidence interval −1.39 to −0.23) for the intervention against the control group.

### 2.7. Risk of Bias

**Figure 3:**
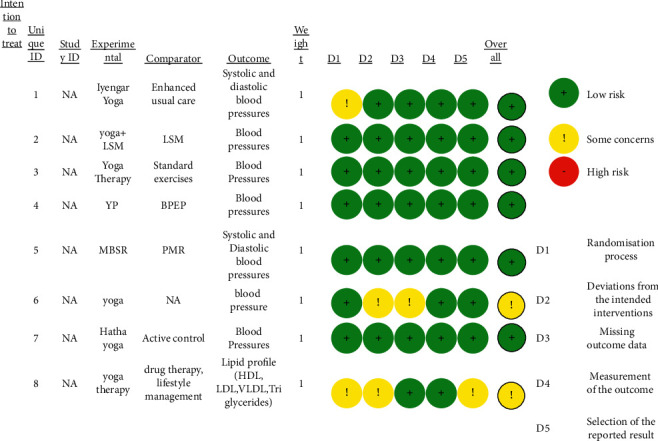
Risk of bias assessment (ROB2 beta v7 by Cochrane).

Risk bias was assessed using the Risk of Bias Assessment Tol ROB2 Beta v7 by Cochrane [10]. The assessment of all 8 articles has been provided in Figure 3. The articles were classified into high, some concerns, and low by the software tool. Two studies were classified as some concerns [11, 12]. This was because one of the studies lacked a proper description for subject recruitment and insufficient data for confounders and follow-up details. Blinding details were also mentioned in only one study where both the participants and the main outcome assessors were blinded [13]. The studies in which blinding is not specified, the risk of bias is possible. Randomisation details are mentioned in all of the studies but only a few studies specify the type of randomisation used [11–18].

## 3. Results

The results of the included studies and their meta-analysis reveal that yoga has a significant role in lowering the blood pressure. The forest plot of the same has been shown in Figure 3 which depicts the results of the meta-analysis. Yoga therapy has shown to influence the systolic pressures (−0.62 standard mean difference, at IV fixed 95% CI (−0.83, (−0.41) more than the diastolic pressures ((−0.81 standard mean difference, at IV random 95% CI (−1.39, (−0.22). The abovementioned values are demonstrated in a narrow confidence interval range signifying the validity and sensitivity of the analysis and true effect. Moreover, the random effects model has used the sample size and standard error for weighing the studies and providing the accurate results. Wherever possible, the intention-to-treat effect has been considered. The meta-analysis also provides a result which states favourable decision for yoga therapy. The studies selected for meta-analysis showed significant heterogeneity making the meta-analysis difficult. The chosen studies were chosen for meta-analysis as they showed homogeneity in the main outcome measures, that is, systolic and diastolic blood pressures. Therefore, we had to use both fixed and random effects model for our meta-analysis. All the studies included in the meta-analysis share almost equal weightage, the majority by Thiyagarajan et al. [18]. The details of the studies included in the review are shown in Table 3.

## 4. Discussion

The possible reasons for the reduction in blood pressures could be due to reduction in vagal tone as a result of relaxation caused due to controlled and slow breathing which is practised in pranayamas [19]. Reduced vagal tone also causes a reduction in heart rate as a result of change in the sympathetic stimulation and a change in the vascular system due to parasympathetic stimulation [19]. Another reason as mentioned by Thiyagarajan et al. could be the “vascular conditioning” effect due to exercises [20]. Exercises produce a shearing force on the internal vasculature and increase the levels and availability of endothelial nitric oxide synthase enzyme which causes vasodilation and reduction in BP [21].

Stress is another factor which has been identified for elevation in blood pressures [4, 5]. There is an increase in the activity of sympathetic nervous system and hypothalamic-pituitary-adrenocortical axis during the time of stressful situations [22, 23]. Stimulation of sympathetic nervous system causes release of norepinephrines, catecholamines, and epinephrines which increases the heart rate and vasoconstriction of the blood vessels [24]. Cortisol is an important regulating factor in BP regulation by controlling sodium retention in the body [25], and salivary alpha amylase is a biomarker for the activity of sympathetic nervous system [26]. There is a significant effect on yoga in reduction of cortisol levels (−2.1 ± 6.0) and salivary alpha amylase levels (−16.4 ± 75.2) in one of the studies included [15]. Sieverdes et al. also found that the levels of salivary amylase were low in the early morning samples. It is referred to as “morning awakening curve” [26, 27].

Out of 8 studies, 5 studies described the yoga asanas and the protocol they followed during the study [12, 15–18]. The common asanas included bhujangasana, setubandhasana, ardhachakrasana, uttanasana, padottanasana, ardhachandrasana, tadasana, shalabhasana, and shavasana. Shavasana was mostly used for relaxation or as the starting pose for asanas. Along with these asanas, some other asanas were also used which have been described in the previous table. The techniques of anulom vilom, pranayama and its variants, and ujjayi (victorious breath) were also used along with yogic postures [12, 16, 17].

The studies in the review had combinations of all the three elements of meditation, posture, and breathing. There was one study which used the mindfulness based stress reduction (MBSR) in comparison with the progressive muscle relaxation (PMR) technique [13]. The MBSR comprised yoga, meditation, and body scan exercises. MBSR has proven to be beneficial based on their statistical analysis.

The outcome measures were not limited to SBP and DBP only. Other outcomes which were studied were heart rate, BMI, waist circumference, and weight [12, 17, 18]. There was a reduction in heart rate in both study and control groups, which was not very significant [15, 17, 18]. The reduction is seen more in Hatha yoga procedure group as reported by Sieverdes et al. (mean difference (−2.7 ± 9.5 for Hatha yoga group and (−0.20 ± 12.1) for control group). Only one study used BMI as an outcome measure and showed minimal changes in the pre- and postintervention statistics [18]. Two of the studies included weight in their outcome measures [12, 18]. The differences in the pre- and postdata were small but significant for the study groups. The weight reduction was higher when the duration of the protocol was 6 months [12]. Only one study took waist circumference as their outcome measure [18]. The difference was not very significant for both the groups but was more for the study group.

Two studies studied the effects of yoga on lipid profile including their high density lipoproteins (HDL), low density lipoproteins (LDL), very low density lipoproteins (VLDL), triglycerides, and total cholesterol (TC) levels [11, 18]. One study solely included the lipid profile values but was included in the review [11]. The main outcomes were not studied in this study but, if excluded, would have biased the review. The study group showed a lowering of the total cholesterol levels as compared to the drug therapy and lifestyle modification. Similar results were found for VLDL and LDL values. There was a significant increase in the HDL values in the study group as compared to the control group. This was seen over a period of 12 months. Another study was done for 12 weeks, using the outcome measures LDL, HDL, TC, and triglycerides. The changes were observed in both study and control groups. The difference was more in the study group as compared to the control group. There was a small reduction in LDL and TC values and a small increase in HDL values [11, 18]. The change was significantly observed in triglyceride levels [18]. The analyses of both articles suggest that a larger change is observed when the duration of protocol is more, and the benefit of lifestyle management is more when it is combined with yoga intervention.

One quasiexperimental study assessed the quality of life as one of the main outcomes [12]. This study was also included to avoid the bias in review. The study group showed a small increase in the quality-of-life scores, whereas a downtrend was observed in the control group as compared to the study groups.

This review includes studies which are majorly RCTs. The meta-analysis result favours yoga intervention. This establishes that there is a positive effect of yoga on prehypertension, and yoga therapy is beneficial in lowering the blood pressure levels. The review has tried to eliminate bias during selection of articles; however, two of the included articles pose a possibility for bias [11, 12]. Eliminating their effects, the conclusions have been drawn. This review has included all possible evidence available on yoga and prehypertension and its effects on blood pressures.

### 4.1. Limitations

Certain studies did not include the direct mention of the word “yoga” or “pranayama,” which have been excluded as a part of screening process, may have biased our review, and reduced the number of articles included in the study. Certain breathing practises which were not labelled as yogic breathing were also excluded. This review has not included the effects of other forms of exercises like tai-chi or qigong, which have emerging evidence on prehypertension and hypertension. The review also has a shortcoming at providing a structured yoga dose due to the lack of proper evidence for the same. Only one article has been identified for the same [17]. A future scope may include a comparison between these forms of exercises and traditional yogic practises.

## 5. Conclusion

This review is the first systematic review and meta-analysis done solely in the topic of prehypertension and yoga. Yoga therapy has been proven beneficial. It has a significant effect on SBP and DBP. It has also proven to be beneficial for reduction in lipid profile when practised for a longer period. Certain asanas, which were found beneficial and used most widely in the majority of the evidence obtained, were bhujangasana, setubandhasana, ardhachakrasana, uttanasana, padottanasana, ardhachandrasana, tadasana, shalabhasana, and shavasana. One of the studies has also used sun salutations (surya namaskar) as a warm-up exercise and has been proven beneficial. Meta-analysis has proven a statistically significant reduction on blood pressures, thereby proving the positive effect of yoga on blood pressures. A need for studies with a proper structured yoga dosage is required in future in this area of research.

## Figures and Tables

**Table 1:** Keywords used: Strategy builder.

Sr. no.	Strategy
1.	Basic keyword yoga with ‘OR'
2.	Basic keyword blood pressure with ‘OR'
3.	Basic keyword prehypertension with ‘OR'
4.	Combined searches with ‘AND'
5.	Time span filter (2010–2021)
6.	Full-text filter
7.	RCT filter

RCT- Randomised control trial.

**Table 2:** Synonyms and keywords.

^*∗*^Yoga	^*∗*^Blood pressure	^*∗*^Prehypertension
Iyengar	Vital sign	Borderline hypertension
Ashtanga	Pressure level	Elevated blood pressure
Astanga	Systolic pressure	Prehypertensions
Asana	Diastolic pressure	Prehypertension
Hatha	Arterial pressure	Prehypertensions
Yogasana	Systolic pressures	Prehypertension
Mind-body therapy	Diastolic pressures	Pre hypertensions
Meditation		Elevated blood pressures
Kriya		
Kundalini		
Anusara		
Kripalu		
Chikitsa		
Bikram		
Pranayama		
Anulom vilom		
Alternate nostril breathing		
Vinyasa		
Mudras		
Mudra		
Ujjayi		

^*∗*^Words highlighted in bold are main keywords.

**Table 3:** Characteristics of studies included.

Sr. no.	Author/year	Sample size	Study population	Intervention description		Frequency		Total time (min)		B.P measurement	
				Study	Control	Study	Control				
1	Thiyagarajan, et al. [18]	192	Age- 20-60 years, without known CVD, SBP 120–139 mmHg, DBP 80–89 mmHg.	DASH diet, aerobic physical activity, body weight management (BMI 18.5–24.9 Kg/m2) + yoga sessions breath-body coordination practice, joint loosening practice, asanas-talasana, ardhakati chakrasana, ardhachakrasana, uttanpadasana, ardhahalasana, pawanmuktasana, sarvangasana, makarasana, bhujangasana, dhanurasana; pranayama (pranav, chandranadi, nadi shuddhi pranayamas), relaxation- kayakriya in shavasana, and shavasana with savitri pranayama.	DASH diet + aerobic physical activity, body weight management (BMI 18.5–24.9 Kg/m2)	3 sessions/week	7 days/week	1620 (mentioned only for yoga group), not reported for control group		Automatic BP monitor	
2	Vrinda hari ankolekar, [12]	102	Participants with prehypertension (AHA criteria)	Yoga session-asanas-tadasana, trikonasana, vajrasana, suptavajrasana, pawanmuktasana, bhujangasana, dhanurasana, parshwakonasana, shalabhasana, padottanasana, vakrasana, shavasana; pranayama, anulom vilom, suryabhedana, chandrabhedana, bhramari, and meditation.	Not reported	First 15 days- 60 min/day; 45 min for 6 days/week	Not reported	7380 (reported only for the study group.)		Sphygmomanometer	
3	Hagins, et al. [17]	84	Age- 21–70 years, SBP between 120 and 159 mmHg, DBP 80–99 mmHg, medically stable on any current medications, BMI 18.5–40 Kg/m^2^	Asanas-bhujangasana, setubandasana, chakravasana, uttanasana, suryanamaskar, veerbhadrasasana, padahastasana, trikonasana, utthita-parsvakonasana, prasarita padottanasana, side stretching, janu shirshasana, shalabhasana, titli asana, shavasana. 1st month: warm up. 2nd month-suryanamaskar, 3rd month- veerbhadrasana Asanas for meditation-sukhasana, ujjayi.	Body weight exercises, stretching, therabands, equipment based.	55 min sessions for 12 weeks. 2 sessions supervised, 3 sessions unsupervised/week.	55 min sessions for 12 weeks. 2 sessions supervised, 3 sessions unsupervised/week.	3300 for both groups.		Ambulatory BP by aneroid sphygmomanommeter.	
4	Hughes et al., [13]	56	Age 30–60 years, unmedicated BP, SBP 120–139 mmHg, DBP 80–89 mmHg.	MBSR-meditation, yoga exercises, body scan exercise. Yoga exercises not mentioned.	16 muscle groups to 4 muscle group relaxation and later relaxation by recall.	8 sessions of 150 min; 45 min 6 sessions/week	8 sessions of 150 min; 45 min 6 sessions/week	4320 for both groups.		Automated oscillometer BP device	
5	Cohen et al., [14]	78	Age 29–69, untreated SBP 130–160 mmHg, DBP less than 100 mmHg.	IY-savasana, supta badda konasana, supta swastikasana, bharadwajasana, pavanamuktasana, adho mukkha viasana, adho mukha swastikasana, adho mukkha savasana	EUC- dietary control classes- lifestyle management classes and active BP control lectures, motivational classes.	1st 6 weeks -twice a week session (70 min), 2nd 6 weeks once a week session+ 25 min DVD based home practice	4–60 min group classes, and 2–30 min individual phone calls.	2310 for IY group+ 300 for EUC		Ambulatory BP.	
6	Sieverdes, et al. [15]	31	Seventh grade students who did not have experience in formalised yoga programmes, nonhypertensive youth.	Hatha yoga-ardha chandrasana, setubandasana, utkatasana, bhujangasana, tadasana, adho mukha shwanasana, upavista konasana, uttanasana, vrikshasana, veerbhadrasana, shawasana pranayama, ujjayi.	Active group- music and art classes according to calendar.	6 weeks 2 session 90 min session, and 6 weeks 3 sessions 90 min, alternately	6 weeks 2 session 90 min session, and 6 weeks 3 sessions 90 min, alternately	2700 for both groups.		B.P monitor machine	
7	Mahesh et al., [11]	88	Prehypertensive SBP 120–139 mmHg, DBP 80–89 mmHg.	Simple yogic exercise	Prescribed drugs with lifestyle modification.	120 min for 2 weeks (supervised), self-performed exercises (unsupervised- rest of the study time).	Not reported	1680 for yoga group.		Not reported	

Sr. no.	Author/year	Sample size	Study population	Intervention description			Frequency			Total time (min)	B.P measurement
				Study	Control	Combo	Study	Control	Combo		
8	Cohen, et al. [14]	137	Willing participants who gave voluntary written consent, age ≥18 years, SBP ≥130 mmHg but less than 160 mmHg.	Yoga group- asanas not mentioned- hatha yoga	BPEP- small group health education, walking programme, 12 nutritional classes + motivational classes.	Yoga classes, nutrition lectures, walking programme. Optional- motivational lectures and home practice	90-minute session biweekly for 1st 12 weeks. Later 12 weeks- community classes of yoga.	6 days a week, (30 min of walk/10,000 steps per day)	Yoga- 2 sessions/week, nutrition- 2 sessions/week, 30 min walking programme.	12960	Ambulatory B.P measurement (machine)

CVD: cardiovascular disease, SBP: systolic blood pressure, DBP: diastolic blood pressure, DASH: dietary approaches to stop hypertension, BMI: body mass index, AHA: American Heart Association, EUC: enhanced usual care, SBP: systolic blood pressure, DBP: diastolic blood pressure, and BPEP: blood pressure education programme.

## Data Availability

The data used to support the findings of this study are available from the corresponding author upon request.
